# Morphometric Magnetic Resonance Imaging Study in Children With Primary Monosymptomatic Nocturnal Enuresis

**DOI:** 10.3389/fped.2018.00103

**Published:** 2018-04-13

**Authors:** Mengxing Wang, Anyi Zhang, Jilei Zhang, Haifeng Lu, Shuai Xu, Zhaoxia Qin, Jun Ma, Xiaoxia Du

**Affiliations:** ^1^Shanghai Key Laboratory of Magnetic Resonance, Department of Physics, School of Physics and Materials Science, East China Normal University, Shanghai, China; ^2^Department of Developmental and Behavioral Pediatrics, Shanghai Institute of Pediatric Translational Medicine, Shanghai Children’s Medical Center, Shanghai Jiao Tong University School of Medicine, Shanghai, China; ^3^MOE-Shanghai Key Laboratory of Children’s Environmental Health, Shanghai Jiao Tong University School of Medicine, Shanghai, China

**Keywords:** nocturnal enuresis, magnetic resonance imaging, structural change, supplementary motor area, precuneus

## Abstract

**Objective:**

Primary monosymptomatic nocturnal enuresis (PMNE) refers to bed-wetting in children who have no other lower urinary tract symptoms and are never dry for more than 6 months. Our previous studies demonstrated that children with PMNE exhibited brain functional abnormalities compared with healthy controls; however, researches on the abnormalities in gray matter were limited. This study aimed to investigate brain structural changes in gray matter of children with PMNE using magnetic resonance imaging (MRI).

**Methods:**

Gray matter volumes (GMVs) and gyrification indices (GIs) were calculated using voxel-based and surface-based morphometry analyses of structural MRI data acquired from 26 children with PMNE and 28 healthy children. To identify between-group differences in gray matter, two-sample *t*-tests were conducted on GMV and GI images separately.

**Results:**

Compared with the controls, children with PMNE showed significantly increased GMVs in the supplementary motor area and medial prefrontal cortex regions (mean GMV in PMNE: 0.54 ± 0.07 l; mean GMV in controls: 0.50 ± 0.06 l) and reduced GIs in the right precuneus (mean GI in PMNE: 25.74° ± 2.34°; mean GI in controls: 27.97° ± 1.79°).

**Conclusion:**

Children with PMNE showed abnormal GMVs in frontal lobe and GIs in precuneus, and these changes might be involved in the pathological mechanism of PMNE.

## Introduction

Enuresis, defined as children aged 5 years or older who wet the bed at least twice a week for three consecutive months, is not induced by any substance/polyuria/general medical condition according to the Diagnostic and Statistical Manual of Mental Disorders (5th edition). Enuresis is common in childhood, and its prevalence was reported as nearly 10% in 7-year-old children and 2% in 12-year-old children (1–3). As the dominant subtype, primary monosymptomatic nocturnal enuresis (PMNE) refers to bed-wetting in children who have no other lower urinary tract symptoms and are never dry for more than 6 months (4, 5). In addition to bed-wetting, children with enuresis usually suffer from low self-esteem, poor school performance, and low life quality, which affect their development (6–9).

The causes of enuresis are mainly related to genetics, nocturnal polyuria, nocturnal detrusor overactivity, high arousal thresholds, and disturbed central nervous system mechanisms (10, 11). Enuretic children can more easily fill their bladder due to increased urine output and decreased bladder available capacity but are more difficult to wake up; involuntary voiding is inhibited mainly by the brainstem and is controlled by some superior regions such as the thalamus, supplementary motor area (SMA), anterior cingulate cortex, and prefrontal cortex (PFC) (12, 13).

In recent years, magnetic resonance imaging (MRI) has been used to study enuresis, revealing significant functional changes between children with and without enuresis. Yu et al. demonstrated that the elevated oxygen extraction fraction values in primary nocturnal enuresis were positively correlated with the difficulty of arousal (14). Furthermore, studies using task functional MRI associated with working memory and response inhibition, respectively, identified that enuretic children exhibited different patterns (15–17). In addition, resting-state functional MRI studies have found that children with PMNE exhibit not only local spontaneous brain activity changes but also brain network alterations, compared with the controls (4, 18, 19).

Furthermore, a few studies focusing on structural changes in enuresis have also been performed. By using diffusion tensor images of children with PMNE, we previously demonstrated that some brain regions related to micturition control, such as the thalamus, medial frontal cortex, and anterior cingulate cortex, exhibited significant microstructural abnormalities (20). Yu et al. found that decreased gray matter (GM) densities may be involved in memory/attention deficits in children with primary nocturnal enuresis (21).

Considering that the function of the human brain is always associated with its structure and researches on the GM abnormalities of PMNE are limited, we hypothesize that these children may have brain structural abnormalities related to micturition control or arousal mechanisms. Examples of these changes include altered gray matter volume (GMV) and folding, and they may be associated with the pathological mechanisms of enuresis. Voxel-based morphometry (VBM) and surface-based morphometry (SBM) analyses, for example, calculating GMV and gyrification index (GI; based on the absolute values of mean curvature of the cerebral cortical surface), are beneficial for measuring structural abnormalities (specifically in GM) in patients without a prior hypothesis (22, 23). Thus, this study used VBM and SBM analyses to evaluate GM brain structural alterations in children with PMNE.

## Materials and Methods

### Participants

In total, 26 children with PMNE and 28 generally age- and gender-matched healthy controls participated in this study (Table 1). All the participants were from primary and junior middle schools in Shanghai and the surrounding areas. The patients were outpatients from Shanghai Children Medical Center who were diagnosed by Doctor Ma. The PMNE inclusion criteria were as follows: bed-wetting during the night but asymptomatic during the day time; frequency of twice per week or more for three consecutive months; never dry for more than 6 months; symptoms not caused by any related diseases or medicine; and no other lower urinary tract diseases. Healthy volunteers, recruited using advertisements, had not wet the bed since 5 years of age. Children with any other neurological diseases (such as attention deficit/hyperactivity disorder and autism), internal implant metals, or claustrophobia were excluded prior to MR scanning. All children achieved IQ test scores greater than 80 (Wechsler Intelligence Scale for Children-Revised) and were right handed. This study was carried out in accordance with the recommendations from the East China Normal University Committee on Human Research Protection. Both the children and their parents signed informed consent forms.

**Table 1:** Demographic and clinical characteristics of the sample.

Measure	PMNE children (*n* = 26)	Control children (*n* = 28)
Age (years), mean ± SD	9.53 ± 1.37	9.50 ± 1.42
Gender (M/F), *n*	14/12	15/13
Years of education, mean ± SD	3.35 ± 1.44	3.18 ± 1.52
Bed-wetting frequency (per week), mean ± SD	5.13 ± 2.02	N/A
Bed-wetting frequency (per night), mean ± SD	1.24 ± 0.57	N/A
Number of patients never waking up for voluntary voiding	12 (*n* = 26)	N/A
Number of patients never waking up after bed-wetting	17 (*n* = 26)	N/A

*F, female; M, male; PMNE, primary monosymptomatic nocturnal enuresis*.

### MRI Protocols

Magnetic resonance imaging data were collected at the Shanghai Key Laboratory of Magnetic Resonance (East China Normal University, Shanghai, China) using a 3.0 Tesla Siemens Trio Tim system (Siemens, Erlangen, Germany). Custom-fit foam pads were placed around the participants’ heads to minimize motion, and a 12-channel head coil was used to scan. Whole-brain anatomical volumes were obtained using a high-resolution T1-weighted three-dimensional magnetization-prepared rapid acquisition gradient echo pulse sequence (repetition time = 1,900 ms, echo time = 3.42 ms, inversion time = 900 ms, field of view = 240 mm^2^ × 240 mm^2^, acquisition matrix = 256 × 256, slice thickness = 1 mm, 192 slices).

### VBM and SBM Analyses

Voxel-based morphometry and SBM analyses were performed using the Computational Anatomy Toolbox (CAT12^1^; Structural Brain Mapping Group, Jena University Hospital, Jena, Germany), which is an extension of Statistical Parametric Mapping software (SPM12^2^; Wellcome Trust Centre for Neuroimaging, University College London, United Kingdom). Briefly, preprocessing included bias correction, normalization to the standard MRI space using the DARTEL-algorithm, and segmentation into GM/whiter matter/cerebrospinal fluid. Considering that the subjects were children rather than adults, preprocessing was optimized based on recommendations in the CAT12 toolbox manual. All the T1-weighted images were initially segmented using a customized tissue probability map (TPM) created with Template-O-Matic Toolbox (TOM8^3^; Imaging Research Center, Cincinnati Children’s Hospital Medical Center, OH, USA) and normalized to a customized DARTEL-template created with SPM12. Customized DARTEL template and TPM can reflect the age and gender of a specific population. Next, data quality was evaluated by displaying one slice for all the images and calculating the sample homogeneity. Finally, the modulated normalized GM images were smoothed with a Gaussian filter [8-mm full width at half maximum (FWHM)]. In addition, total intracranial volume was estimated for further analyses.

The central cortical surface for left and right hemisphere in each participant was created on the basis of the tissue segmentation using a fully automated SBM method provided by the CAT12 toolbox (23). GIs were extracted based on the absolute mean curvature from each vertex of the cortical surface mesh, and then GI images were smoothed with a FWHM kernel of 15 mm.

### Statistics Analyses

Smoothed GM and GI images across all the participants (26 patients and 28 controls) were entered into group whole-brain analysis. Two-sample *t*-tests based on general linear model implemented in SPM12 were conducted to detect which brain regions showed between-group differences of GMV and cortical folding separately, using age and gender as covariates. In GMV analysis, total intracranial volume was also included as a covariate to avoid different brain sizes confounding the results, and threshold masking was set to an absolute value of 0.2. All the statistical results were assigned thresholds at voxel-level uncorrected *p* < 0.005 and cluster-level FWE corrected *p* < 0.05.

In addition, the GMV and the GI in the peak coordinates across all the participants were extracted from the GM images and the GI images, respectively, and the peak coordinates were acquired from the results of whole-brain analysis. The mean GMV and GI of each group were calculated.

## Results

**Figure 1:**
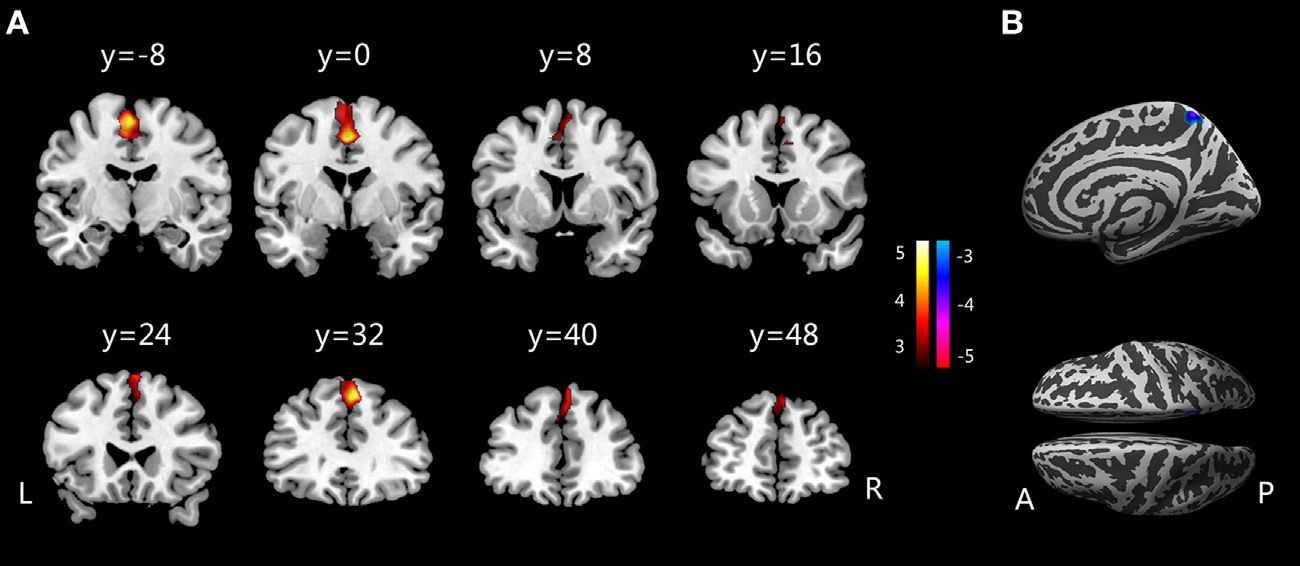
**(A)** Increased gray matter volumes in children with PMNE compared with healthy controls. **(B)** Decreased gyrification indices in children with PMNE compared with healthy controls. A, anterior; L, left; P, posterior; PMNE, primary monosymptomatic nocturnal enuresis; R, right. The color scale indicates the *t*-values.

Whole-brain GMV analysis revealed a cluster of higher GMVs in children with PMNE compared to controls. The cluster included the SMA and medial PFC regions. GIs in the right precuneus of children with PMNE were significantly reduced compared with those in healthy controls. Only clusters surpassing thresholds at voxel-level uncorrected *p* < 0.005 and cluster-level FWE corrected *p* < 0.05 are shown in Table 2 and Figure 1. The mean GMVs of SMA/PFC cluster were 0.54 ± 0.07 l (PMNE) and 0.50 ± 0.06 l (controls); the mean GIs of precuneus were 25.74° ± 2.34° (PMNE) and 27.97° ± 1.79° (controls).

**Table 2:** Significant differences in the GMVs and GIs of children with PMNE and healthy children.

Brain areas	Cluster size	MNI coordinates			Peak T value	*P*_cluster_
		*x*	*y*	*z*		
**GMVs increased in children with PMNE than healthy controls**						
Bilateral medial frontal gyri	4,219	5	−5	60	5.00	0.001
Bilateral superior frontal gyri		6	33	51	4.72	

**GIs decreased in children with PMNE than healthy controls**						
Right precuneus	557	6	−51	61	3.95	0.029

*GI, gyrification index; GMV, gray matter volume; P_cluster_, cluster-level FWE corrected p value; x, y, and z correspond to the peak activated voxel coordinates; PMNE, primary monosymptomatic nocturnal enuresis*.

## Discussion

This study assessed changes in brain GM in children with PMNE using VBM and SBM analyses, revealing that these children exhibited significantly altered GMVs and GIs in their SMA/medial PFC and precuneus regions.

Compared with the healthy controls, children with PMNE showed increased GMVs in their SMA and medial PFC regions. The SMA plays important roles in controlling movement, especially in preparation for voluntary actions (24), and contributes to pelvic floor and urethral sphincter contraction (25, 26). The SMA was also found to be strongly connected with the thalamus (27), which is essential for switching between sleep and wakefulness and relaying sensory and motor signals to the cerebral cortex for further processing and is activated during bladder voiding initiation and filling (12, 28). Bladder filling or voiding is controlled not only by the midbrain and brainstem but also by superior regions. The SMA, as reviewed by Griffiths, is associated with the emotional aspects of bladder control during the storage phase (29). When a person had an urgent need to void during contraction of the urethral sphincter to delay leakage, the SMA would receive signals relayed *via* the thalamus to evoke “urgency” and relax the bladder to prevent incontinence (12, 24). The SMA in enuretic children was activated with urgency during bladder filling in their sleep, but these children failed in their attempt to tighten the urethral sphincter, ultimately wetting the bed.

The PFC is a part of the frontal lobe located anterior to the SMA and has been associated with executive control, decision-making, and controlling voluntary micturition (12). The medial PFC was deactivated upon bladder filling and activated during voiding (26, 30), and people with GM lesions in their medial frontal cortex suffered from micturition disturbances (31). Our results showed that GMVs in children with PMNE were increased in the medial PFC. This region predominantly has an inhibitory effect on controlling micturition in the brain–bladder network, as proposed by Griffiths and Fowler (12, 32). In the normal continence mechanism, the medial PFC connects with the periaqueductal gray directly and indirectly and may enhance inhibition of the brainstem switch. Thus, abnormalities in the medial PFC in children with PMNE may influence their decision to void at night. Furthermore, this result was supported by our previous finding using resting-state functional MRI that decreased amplitude of low-frequency fluctuation and regional homogeneity were present in the medial frontal lobe in children with PMNE (18). Yu et al. had reported that the GM density of enuretic children changed when compared with healthy children in the dorsolateral PFC as well. Therefore, the altered SMA and medial PFC volumes in children with PMNE were in accordance with reported deficits in urinary continence and may be involved in the pathological mechanism of enuresis (18, 26, 29, 31).

Children with PMNE showed decreased GIs in their precuneus, which was consistent with our previous finding that PMNE children exhibited altered regional homogeneity in their precuneus compared with healthy controls (18). Children with PMNE are considered “deep but poor” sleepers due to their high arousal thresholds and frequently disturbed sleep (10). These children are very difficult to wake from sleep and do not void involuntarily during the day. Thus, it is apparent that disturbed sleep and arousal mechanisms exist in PMNE children, and they may not be switched between sleep and wakefulness conscious states (14, 33). The precuneus is regarded as a key brain area for conscious information processing (34, 35). Previous studies found that the precuneus became less active in decreased conscious states, such as sleep and vegetative states (36), and a study on vegetation also reported that the precuneus was reactivated once consciousness returned (37). In response to full bladder sensations, normal children awaken from sleep to avoid wetting bed, while enuretic children often fail to do so, which is attributed to their arousal deficit. Therefore, we considered that structural alterations in the precuneus of children with PMNE might implicate their inability to switch from sleep to wakefulness when confronted with a full bladder at night. Although there might be arousal deficits in the patients of this study, considering the lack of support from sleep/arousal studies, it needs more evidence to demonstrate the connections between abnormal precuneus and their difficult arousal.

While we found significant structural alterations in children with PMNE, our study has several limitations. First, the sample size of children with PMNE was relatively small, and our results should be verified using a larger sample in a subsequent study. Furthermore, it is known that the prevalence rate of PMNE decreases with age (1), and 2–3 years following study would be useful to investigate abnormal development in the brains of children with PMNE (38, 39).

In conclusion, this study evaluated the structural abnormalities of GM in children with PMNE using VBM and SBM, and the alterations in children with PMNE were mainly located in the SMA/medial PFC and precuneus regions. The underlying pathophysiological relations between these alterations in GM and clinical symptoms in PMNE (e.g., deficits in urinary continence, high arousal threshold) need to be further clarified.

## Ethics Statement

This study was carried out in accordance with the recommendations from the East China Normal University Committee on Human Research Protection. Both the children and their parents signed informed consent forms.

## Author Contributions

XD, MW, and JM designed the study. MW, AZ, HL, SX, and ZQ acquired the data. MW and JZ analyzed the data. MW and XD interpreted the data and drafted the manuscript. XD and JM revised the manuscript.

## Conflict of Interest Statement

The authors declare that the research was conducted in the absence of any commercial or financial relationships that could be construed as a potential conflict of interest.

## Footnotes

^1^http://dbm.neuro.uni-jena.de/cat/ (Accessed: May 27, 2017).

^2^http://www.fil.ion.ucl.ac.uk/spm/software/spm12 (Accessed: May 27, 2017).

^3^http://dbm.neuro.uni-jena.de/software/tom (Accessed: May 27, 2017).
